# Formation of Hydrogen Sulfide from Cysteine in *Saccharomyces cerevisiae* BY4742: Genome Wide Screen Reveals a Central Role of the Vacuole

**DOI:** 10.1371/journal.pone.0113869

**Published:** 2014-12-17

**Authors:** Gal Winter, Antonio G. Cordente, Chris Curtin

**Affiliations:** 1 School of Biomedical and Health Sciences, College of Health and Science, University of Western Sydney, Parramatta, NSW, Australia; 2 The Australian Wine Research Institute, Adelaide, SA, Australia; 3 Centre for Microbial Electrosynthesis (CEMES), University of Queensland, Brisbane, Queensland, Australia; Louisiana State University Health Sciences Center, United States of America

## Abstract

Discoveries on the toxic effects of cysteine accumulation and, particularly, recent findings on the many physiological roles of one of the products of cysteine catabolism, hydrogen sulfide (H_2_S), are highlighting the importance of this amino acid and sulfur metabolism in a range of cellular activities. It is also highlighting how little we know about this critical part of cellular metabolism. In the work described here, a genome-wide screen using a deletion collection of *Saccharomyces cerevisiae* revealed a surprising set of genes associated with this process. In addition, the yeast vacuole, not previously associated with cysteine catabolism, emerged as an important compartment for cysteine degradation. Most prominent among the vacuole-related mutants were those involved in vacuole acidification; we identified each of the eight subunits of a vacuole acidification sub-complex (V_1_ of the yeast V-ATPase) as essential for cysteine degradation. Other functions identified included translation, RNA processing, folate-derived one-carbon metabolism, and mitochondrial iron-sulfur homeostasis. This work identified for the first time cellular factors affecting the fundamental process of cysteine catabolism. Results obtained significantly contribute to the understanding of this process and may provide insight into the underlying cause of cysteine accumulation and H_2_S generation in eukaryotes.

## Introduction

The concentration of free intracellular cysteine is tightly regulated in eukaryotic and prokaryotic cells, serving two opposing homeostatic requirements. Cysteine concentration must be sufficient for synthesis of proteins and other essential sulfur containing molecules such as glutathione (GSH) and coenzyme A, while on the other hand cysteine concentration must be kept below the threshold of cytotoxicity [1]. The toxicity of excess cysteine was demonstrated in yeasts [2], [3] and animal models [4], leading to growth inhibition [3], endoplasmic reticulum (ER) stress [2] and is associated with various diseases including Parkinson's and Alzheimer's [5]. A key product of cysteine catabolism is hydrogen sulfide (H_2_S) [1], [6], [7], increasingly recognised to be a powerful regulator of many aspects of cell physiology (Reviewed in: [8], [9]–[12]). Still, despite the increasing interest in H_2_S generated from cysteine, fundamental questions regarding regulation of cysteine homeostasis remain to be answered.

H_2_S generation is evolutionarily conserved in all three kingdoms of life [7]. In mammals, endogenous H_2_S is presumed to be generated through the catabolism of cysteine by two cytosolic enzymes cystathionine β-lyase (CBS) and cystathionine γ-lyase (CSE) [1], [6], [9], [13]–[15]. Regulation of expression and activity of these enzymes remain to be elucidated. Consistent with the evolutionary conservation of cysteine catabolism, mammalian CBS- and CSE-encoding genes are similar to those of yeast and bacteria [16]–[19]. Predicted yeast and human CBS proteins share 72% similarity and these have significant similarity to the predicted rat CBS protein and bacterial cysteine synthase [16]. Expression of the gene encoding a human CBS in yeast was able to recover cysteine-auxotrophy caused by deletion of the yeast native CBS [16]. The predicted yeast CSE product was also found to be closely related to rat and *E. coli* CSE [19]. These similarities reinforce the utility of *S. cerevisiae* as a model eukaryotic system to explore cellular homeostasis of cysteine and the catabolism of cysteine to release H_2_S. In this paper we describe a genome-wide survey using a haploid EUROSCARF *S. cerevisiae* gene deletion library to shed light on the cellular processes influencing cysteine catabolism.

## Materials and Methods

### Reagents, yeast strains and media

Chemical reagents were obtained from Sigma-Aldrich. The *S. cerevisiae* strains used in this study include BY4742 (*MATα/his3Δ1/leu2Δ0/lys2Δ0/ura3Δ0)* [20] and the gene deletion collection in the same genetic background, purchased from EUROSCARF (Frankfurt, Germany). Precultures were grown in YPD medium to be subsequently inoculated into microtiter plates or shake flasks filled with chemically defined medium, based on Winter et al. [21] with the following modifications: 100 g/L sugars (50 g/L glucose, 50 g/L fructose), addition of auxotrophic requirements (20 mg/L uracil, 20 mg/L histidine, 60 mg/L leucine and 30 mg/L lysine), and exclusion of cysteine from the media. Where specified cysteine was supplemented at 500 mg/L and adenine supplementation was 20 mg/L.

### High-throughput H_2_S screening

Screening was performed using a novel high-throughput method that estimated H_2_S excreted in the growth medium during fermentation and allows generation of H_2_S production profiles [22]. For follow-up experiments, H_2_S released during fermentation was detected in the shake-flask headspace using silver nitrate selective gas detector tubes (Komyo Kitagawa, Japan), as described in [23].

### Genome-wide screening for genes involved in cysteine catabolism to H_2_S

Cultures of strains in the *S. cerevisiae* BY4742 EUROSCARF deletion collection were pre-grown to stationary phase, each in 200 µl YPD with agitation (150 rpm) in a microtiter plate. The preculture was inoculated at 20 µl into 180 µl of medium supplemented with cysteine and 10% (v/v) of either H_2_S detection mix [22] including 100 mM citric acid and 5 mg/ml of methylene blue, hereafter referred to as MB samples or water, referred to as control samples. Each plate included a well with the wild-type strain (BY4742) as a control in addition to un-inoculated wells to account for medium contamination and non-enzymatic degradation of cysteine to H_2_S. Cultures were inoculated into a microtiter plate in four equal blocks (wells A1 to D6, A7 to D12, E1 to H6 and E7 to H12), representing four replicates of each strain. Following inoculation, microtiter plates were covered with Breath-Easy membranes (Astral Scientific, Australia) and were incubated at 28°C. Optical density measurements at 600 and 663 nm wavelength were carried out at intervals of three hours, for 36 hours. Inoculation and assay monitoring were handled robotically (Freedom EVO 150, Tecan, Männedorf, Switzerland; Cytomat Automated Incubator, ThermoFisher Scientific, Massachusetts, USA). Where specified, repeat experiments were conducted manually.

Data analysis was carried out using a custom script written in R [24], that constructs growth and H_2_S production curves for each strain as well as performing statistical analysis of replicates. Strains were assessed for ability to produce H_2_S via cysteine catabolism, based on the amount of H_2_S detected using the MBR method for MB samples and their ability to form biomass relative to the wild-type strain as measured in the control samples. A calculation of the minima value of the MB decolourisation curve minus the maxima value of the growth curve was used to classify strains as ‘low’ or ‘high’ H_2_S producers. Strains with scores greater than 1 and lower than 2.5 were classified as ‘low’, while strains with a score smaller than one were defined as ‘high’. As the initial OD of the MB supplemented culture was 2.5–2.7, scores above 2.5 were due to growth defects. Classification was then verified against the control wild type strain in each plate. Strains classified as either low or high producers were verified from follow-up micro-fermentation analysis. Cellular processes identified as important for cysteine catabolism to H_2_S were evaluated for statistical significance (P-value <0.01) with the GO Term Finder program of the *Saccharomyces* Genome Database (http://www.yeastgenome.org/cgi-bin/GO/goTermFinder.pl).

### Generation of respiratory deficient petite cells

Cultures of AWRI1522 (*Δhis3, Δleu2, Δtrp1, Δlys2 mat α*) were grown in YPD with shaking (100 rpm) at 25°C. Mitochondrial mutants (AWRI1520, *mat α, Δhis3, Δleu2, Δtrp1, Δlys2, rho –ve*) were isolated by treating cells for 8 hours in synthetic complete medium (0.17% (wt/vol) yeast nitrogen base without amino acids, 0.5% (wt/vol) ammonium sulfate, 2% (wt/vol) glucose) containing 10 µg/ml ethidium bromide. Cells were then diluted in water and, due to their inability to perform aerobic metabolism and utilise glycerol as a carbohydrate source, mitochondrial mutants were revealed by petite colony growth on YPDG (1% w/v yeast extract, 2% w/v peptone, 3% w/v glycerol and 0.1% w/v glucose) [25]. Loss of respiratory function in these petites was then confirmed by their inability to grow on YPG media (1% w/v yeast extract, 2% w/v peptone, 3% w/v glycerol).

### *In-vivo* V-ATPase downregulation

Cells were grown at 28°C with agitation (150 rpm) in chemically defined medium supplemented with cysteine until mid-log phase (OD 0.5). While producing H_2_S, cells were centrifuged and washed with distilled water prior to transfer into chemically defined medium containing 0.112 M glucose or 0.112 M galactose as carbon source, with or without cysteine supplementation. Cells were then incubated with shaking (150 rpm) at 28°C. Following 15 hours of incubation glucose was added to a final concentration of 0.112 M in medium previously containing galactose as a sole carbon source. Cysteine-generated H_2_S was measured throughout the assay using H_2_S detection tubes. Biomass formation was monitored by measuring the absorbance at 600 nm wavelength.

## Results

### Screen background and methodology

**Figure 1:**
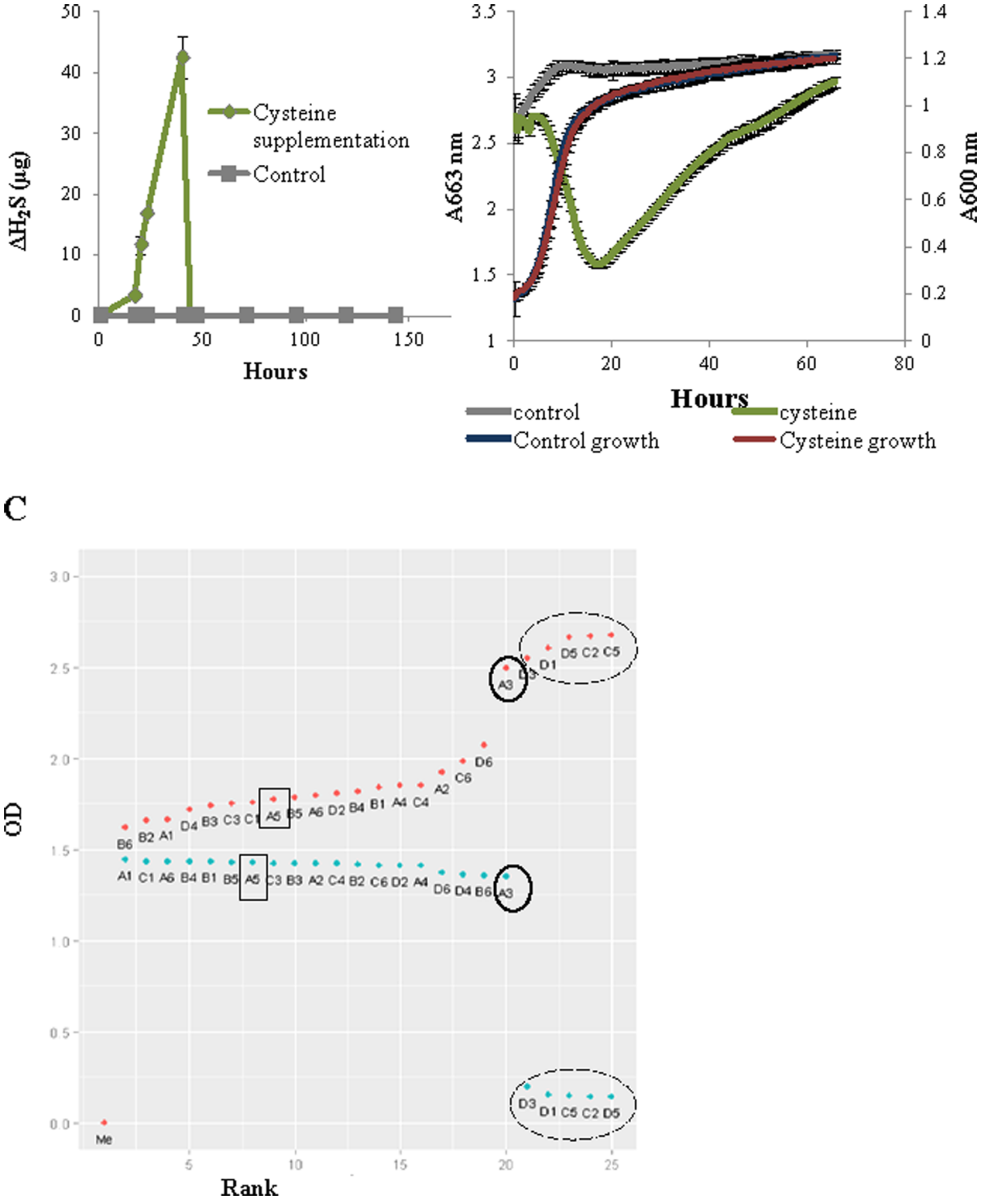
Cysteine catabolism to release H_2_S. Screening process A H2S production profile generated during a shake flask (200 ml) growth of strain BY4742 with or without cysteine supplementation to the medium. H2S was measured using lead acetate detection tubes. Error bars represent standard deviation of triplicate experiments. B H2S production profile and growth curves generated during micro-scale (200 µl) growth of strain BY4742. H2S formation was measured through the degradation of methylene blue measured at 663 nm and biomass formation measured at 600 nm with and without cysteine. Experiments were carried out in a microtiter plate. Error bars represent the standard deviation of quadruplicate experiments. C Illustrative graph of cysteine-generated H2S screen analysis. Graph summarises two replicative microtiter plates assays carried out using cysteine-supplemented media. One measured for growth at 600 nm (blue circles) and the second measured for H2S detection dye degradation at 663 nm, as an indication for H2S formation (red circles). Each plate was inoculated in quadruplicates and data here represents the average of each replicate, labelled with the first replicate well position. Yeasts were ranked based on their methylene blue decolouration measurements (low value indicates dye discolouration and H2S formation). An example of a mutant strain that did not produce H2S while being able to grow under the experimental conditions is circled. Data highlighted in a dashed circle represents either empty wells or strains that were not able to grow. Each plate contained quadruplicates of the background strain (highlighted in rectangle).

While developing a novel high-throughput assay for H_2_S detection [22], we noted that *S. cerevisiae* laboratory strain BY4742 did not produce detectable concentrations of H_2_S when grown in a chemically defined medium. In the current work, after supplementation of medium with cysteine, we detected high concentrations of H_2_S, suggesting this H_2_S was generated by BY4742 solely from cysteine catabolism (Fig. 1). Fig. 1b shows the discolouration of H_2_S detection-dye when cysteine was added to the medium, indicative of H_2_S formation. Notably, H_2_S formation occurred during yeast logarithmic phase of growth and cysteine addition did not affect biomass formation (Fig. 1b).

Because detectable H_2_S in the cysteine supplemented culture of BY4742 is derived from cysteine catabolism, it provides a useful marker to identify genetic factors influencing this cellular process. It was therefore used in a genome-wide screen of *S. cerevisiae* deletion strains [26] to identify cellular processes involved in cysteine catabolism and H_2_S formation. H_2_S production was normalised against biomass formation by plotting the minima for each strain in the assay plate, supplemented with H_2_S detection dye (indicative of maximum H_2_S production) against the maxima for each strain in the growth plate (indicative of maximum biomass formation) (Fig. 1c). Deletants able to grow under the experimental conditions were classified as low or high producers of H_2_S from cysteine based upon their H_2_S minima value in the assay plate, in comparison to the wild-type strain. Fig. 1c shows an example of a strain classified as low producer of H_2_S from cysteine (see well A3, circled).

### Genome-wide classification of mutants that lead to low or high H_2_S production from cysteine

A total of 226 deletion strains displayed differences in their H_2_S accumulation profiles compared to wild type. These were classified as low (188 strains) and high (38 strains) producers of H_2_S from cysteine. Strains were grouped according to gene ontology of the encoded gene product, as defined in the *Saccharomyces* Genome Database (www.yeastgenome.org), using Gorilla [27]. Most deletions that impacted on H_2_S production were for genes from one or more of nine functional groups (Table 1, a comprehensive list is available at S1 Supporting Information). Interestingly, in most cases genes within a group displayed the same trend with respect to H_2_S production (where deletions led to either low or high H_2_S production), indicative of the specificity of the cellular processes involved in catabolism of cysteine to H_2_S. Functional groups of deletions causing high H_2_S production included mitochondrial iron-sulfur homeostasis, mitochondrial translation, and cellular response to ionic iron. Deletions causing low H_2_S production were associated with purine base metabolism process, cellular aromatic compound metabolic process, vacuole acidification, vesicle trafficking to the vacuole, and translation and RNA processing functional groups. Selected deletants from the main functional groups implicated in this screen were further verified using H_2_S detection tubes for an independent H_2_S measurement (S2 Supporting Information). All deletants identified as low H_2_S producers produced significantly lower amounts of H_2_S in comparison to the wild type. For the high producers – H_2_S production was significantly increased during growth of three deletants, one deletants did not show significant difference in H_2_S production in comparison to the wild type and an additional deletants produced significantly lower amounts of H_2_S compared to the wild type. The method's limitation in identification of high H_2_S producers is discussed below. Still an 80% successful validation rate (100% for the low producers) as well as the multiple positive confirmations for major processes implicated in screen reinforces the findings of this work.

**Table 1:** Cellular processes influencing cysteine catabolism identified by H_2_S production phenotype.

Functional group/cellular process	No. of genes	H_2_S production phenotype^*^
Vacuole related	37	Low
Purine base metabolic process	19	Low
Cellular aromatic compound metabolic process	11	Low
Translation	32	Low
RNA Processing	22	Low
Mitochondria related	28	High/Low
Mitochondrion translation	9	High
Cellular response to iron ion	2	High
Unknown function	22	High/Low
Miscellaneous	64	High/Low

**Figure 2:**
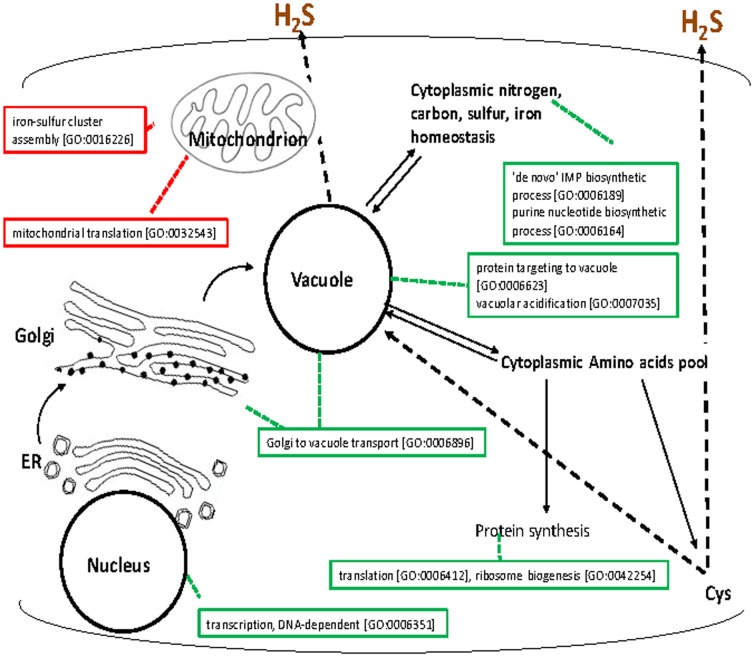
Overview of the cellular processes influencing cysteine catabolism to release H_2_S in *S. cerevisiae*. The figure depicts cysteine catabolism cellular pathway with known and putative transport routes that may mediate the cysteine flux. Boxed annotations indicate some of the cellular processes identified as important for cysteine catabolism to H_2_S and evaluated for statistical significance (P-value <0.01) with the GO Term Finder program of the *Saccharomyces* Genome Database (http://www.yeastgenome.org/cgi-bin/GO/goTermFinder.pl).

Results of this screen provide insight into metabolic and regulatory networks that influence formation of H_2_S from cysteine. A schematic representation of the data is provided in Fig. 2.

### Role of the mitochondrion in catabolism of cysteine to release H_2_S

**Figure 3:**
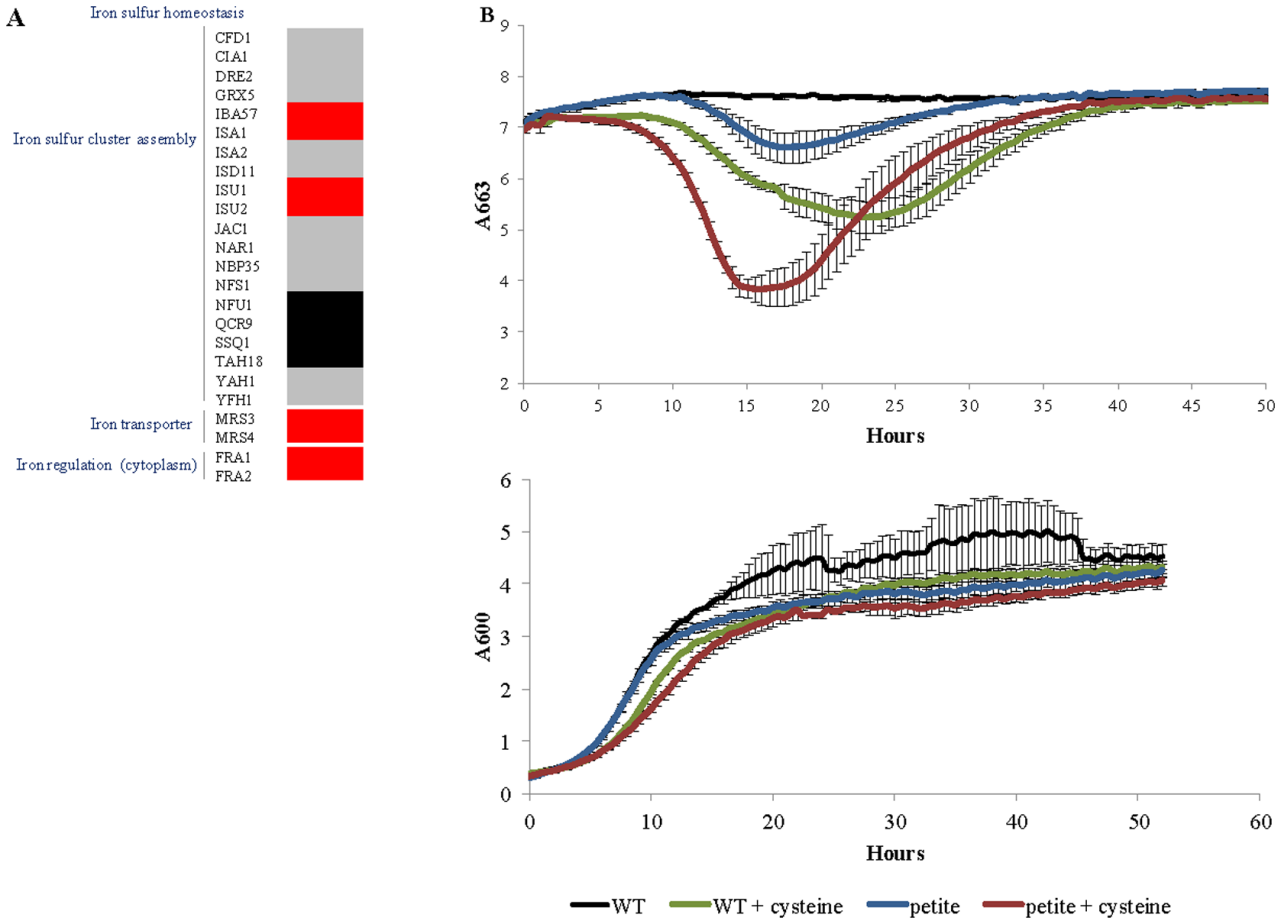
Influence of mitochondrial gene deletions on cysteine catabolism to release H_2_S. **A** Representation of genes involved in cellular iron-sulfur homeostasis with indication of inviable mutants (grey), mutants that lead to ever-secretion of H_2_S (red) and mutants that do not affect H_2_S release from cysteine (black). **B** Extracellular release of cysteine-generated H_2_S (upper graph) and growth curves (lower graph) for *wt* and *petite* cells lacking respiratory function, with and without cysteine supplementation. H_2_S formation was measured at 663 nm using the MBR method growth was measured at 600 nm. Experiments were carried out in a microtiter plate. Error bars represent the standard deviation for quadruplicate experiments.

A large number of deletants affected in mitochondrial function generated high concentrations of H_2_S from cysteine relative to the wild-type strain; none generated H_2_S at detectable concentrations in the absence of cysteine. Amongst these H_2_S producers were strains with deletions in genes involved in mitochondrial maintenance of iron-sulfur (Fe-S) homeostasis (Fig. 3a). ‘Maintenance of Fe-S homeostasis’ includes genes involved in Fe-S cluster synthesis, iron transport into mitochondria, and a cytosolic protein complex involved in regulating transcription of the iron regulon in response to mitochondrial Fe-S cluster synthesis. Whilst for most genes involved in these processes null mutation leads to inviability [28], there were some viable deletants from each sub-group, and these were ‘high’ producers of H_2_S from cysteine (Fig. 3a), highlighting the role of Fe-S homeostasis in cysteine catabolism to release H_2_S.

An additional class of deletants overproducing H_2_S from cysteine were those impaired in mitochondrial translation (*Δimg2, Δrml2, Δmrps35, Δmrpl20, Δmrpl4, Δmrpl24, Δmrpl10*), suggesting that *de novo* synthesis of mitochondrial proteins is important for cysteine catabolism. The connection between mitochondrial function and H_2_S generation from cysteine was further examined using respiratory deficient petite mutants. These strains were shown to produce considerably higher concentrations of cysteine-derived H_2_S relative to the wild-type strain, and to produce detectable amounts of H_2_S even in the absence of cysteine (Fig. 3b). These results reinforce the importance of mitochondrial function in H_2_S production resulting from cysteine catabolism. The small, but nonetheless detectable, levels of H_2_S in the absence of supplemented cysteine may be due to catabolism of endogenous sources of this amino acid. Further experimental work is required to test this.

### Low levels of production of H_2_S from cysteine

**Figure 4:**
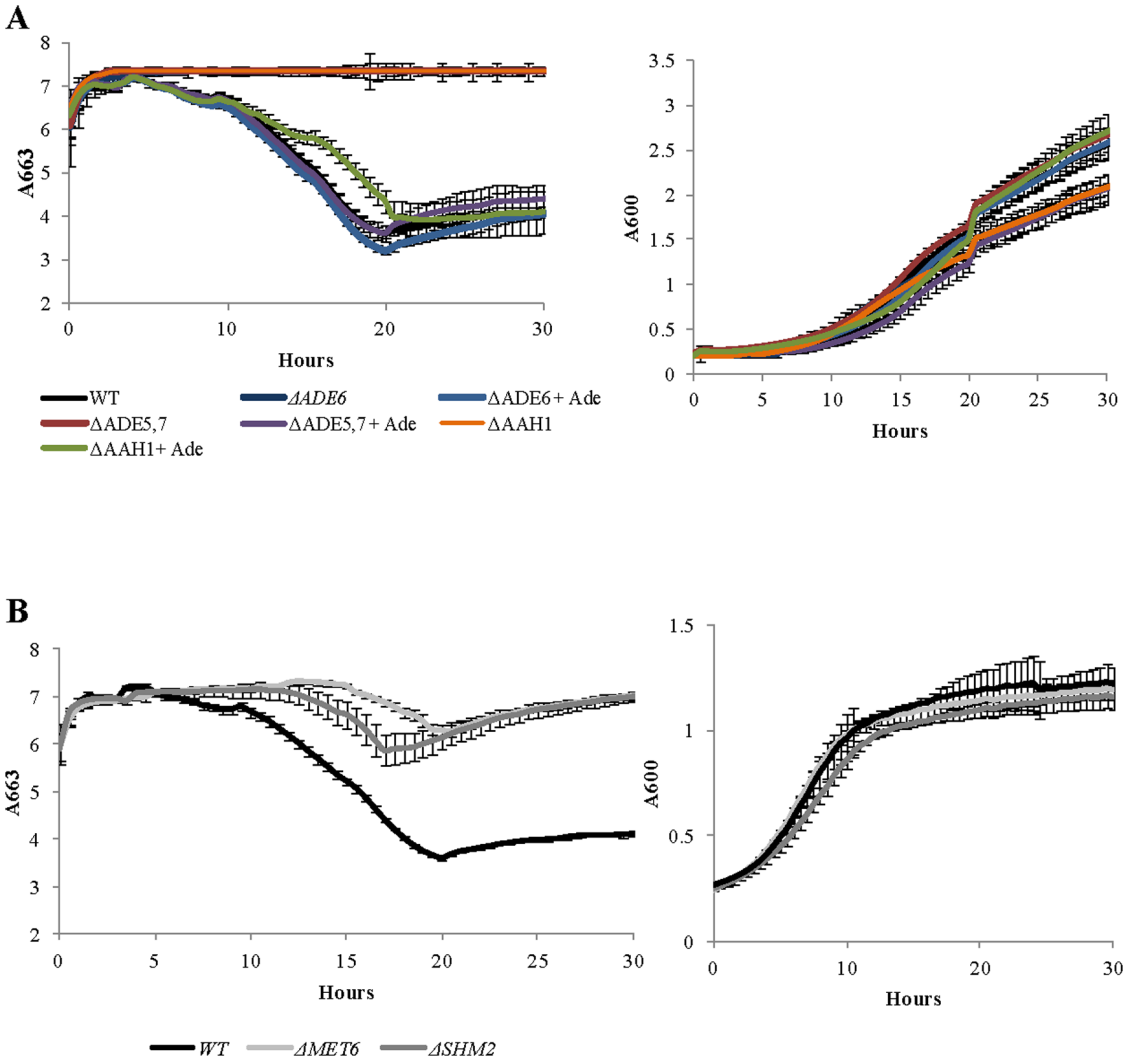
Influences of mutants in purine metabolism on cysteine catabolism to H_2_S. **A** Extracellular release of cysteine-generated H_2_S (left panel) and growth curves (right panel) from *wt* and mutants in purine biosynthesis pathway, with and without adenine supplementation. **B** Extracellular release of cysteine-generated H_2_S (left panel) and growth curves (right panel) from *wt* and mutants in folate derived one-carbon metabolism. H_2_S formation was measured at 663 nm using detection-dye; discolouration of the dye represents release of H_2_S. Experiments were carried out in a microtiter plate. Error bars represent the standard deviation for quadruplicate experiments.

A total of 188 deletants were shown to release less H_2_S into cysteine-supplemented medium than the wild-type strain. Included in this number were deletions of genes involved in purine base metabolism and, more specifically, in the *de novo* synthesis of inosine monophosphate (IMP); *Δade1, Δade5,7, Δade6, Δade8, Δado1*, *Δaah1, Δapt2*. Strains with these deletions also grew significantly slower than the wild type, with or without cysteine addition. It is worth noting that mutants for purine metabolism have an auxotrophic requirement for adenine [29]. The medium used for the screen described here did not contain adenine, however mutants were still able to reach late logarithmic phase of growth (Fig. 4). Upon supplementation of adenine, mutants were able to grow and catabolise cysteine to produce H_2_S at a rate similar to the wild type (Fig. 4). Purine synthesis is linked to folate one-carbon-group metabolism and the metabolism of methionine [30]. Interestingly, deletion of genes involved in these pathways (*Δshm2, Δmet6*) resulted in low H_2_S release from cysteine, although these strains were able to grow at a rate similar to the wild type (Fig. 4).

### Deletants affected in vacuolar function

**Figure 5:**
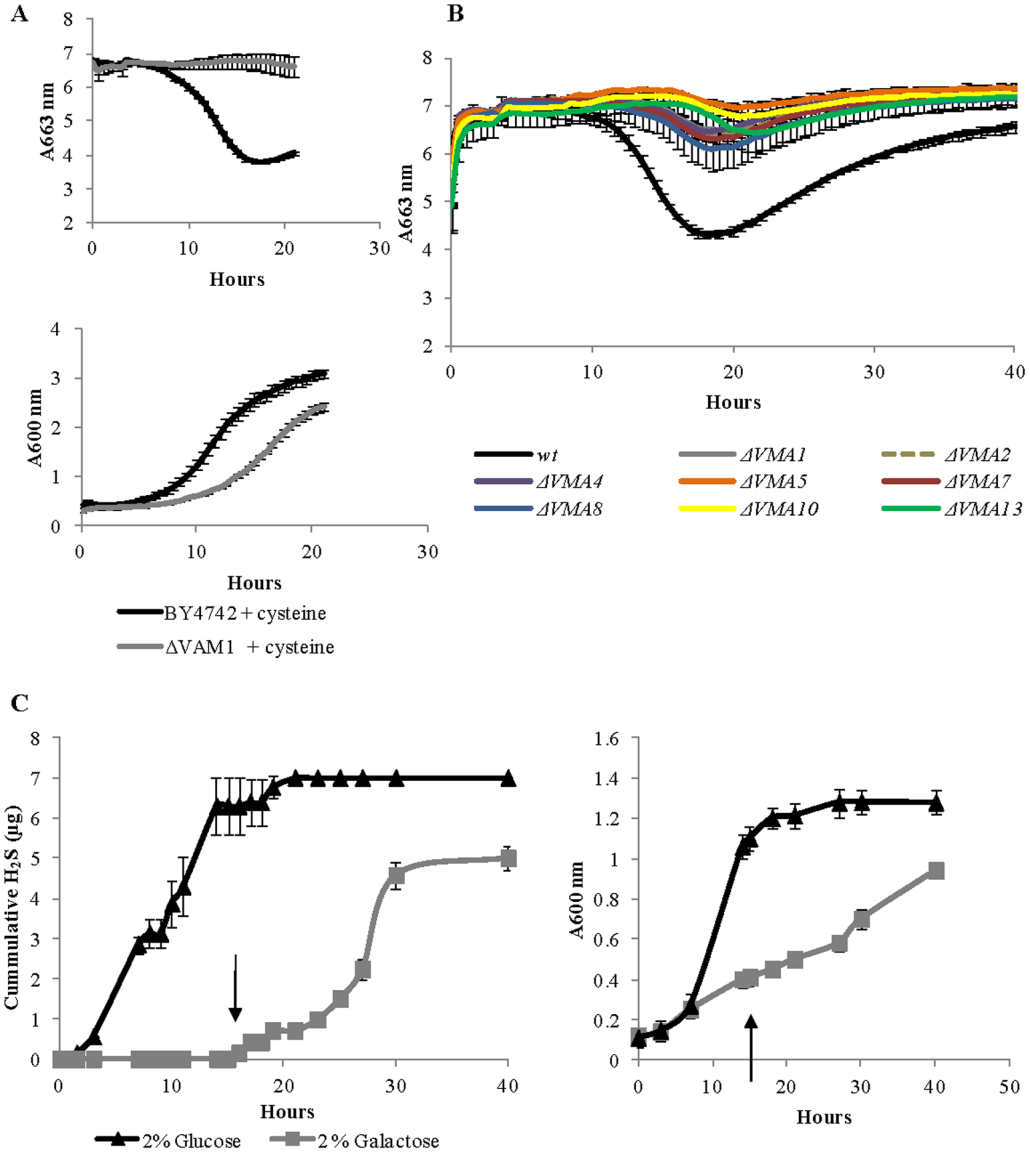
Central role of the vacuole in cysteine catabolism to H_2_S. **A** Extracellular release of cysteine-generated H_2_S (upper panel) and biomass formation (lower panel) profiles of *wt* and mutant in vacuole biogenesis (*Δvam1*). Experiments were carried out in quadruplicates. **B** Extracellular release of cysteine-generated H_2_S for mutants deleted in each of the sub-units comprising V_1_ of the V-ATPase complex. Experiments were carried out in quadruplicates. **C** *in-vivo* downregulation of the yeast V-ATPase complex through nutrient limitation. H_2_S generation profile measured with H_2_S detection tubes (left panel) and biomass formation profile (right panel) for BY4742 cells grown with glucose or galactose as carbon source. An addition of glucose to a final concentration of 0.112 M was made to the galactose medium at the time indicated by arrows.

A large group of 39 deletants impaired in the release of H_2_S from cysteine-supplemented medium included those affected in vacuolar function and vesicle transport to the vacuole. Amongst these were deletions in genes involved in vacuole acidification, transport to the vacuole and vesicle fusion (Table 2). Cells deficient in vacuole biogenesis (*Δvam1*) did not produce H_2_S when supplemented with cysteine. Moreover, cysteine supplementation resulted in growth inhibition for this deletant (Fig. 5a). Notably, while the mutant grew more slowly than wild-type, comparison of H_2_S production at the same growth stage shows H_2_S accumulation by the wild type strain and not by the deletant.

**Table 2:** Vacuole function impaired deletants classified as low H_2_S producers.

Functional Group	Deletants
Vacuole acidification	*Δvps3, Δvma3, Δvps45, Δvps16, Δvps34, Δvma22, Δvph2, Δmeh1, Δvma13, Δvma1, Δvma2, Δvma4, Δvma7, Δvma10, Δvma13, Δvma21*
Transport to the vacuole	*Δccz1, Δypt10, Δvps52, Δvma3, Δvma8, Δvps45, Δmon1, Δcog1, Δgtr2, Δavt7, Δmeh1, Δvts1, Δvma13, Δshp1, Δsym2, Δatg18*
Vesicle fusion	*Δvam7, Δpep12, Δvps45, Δvts1, Δyck3, Δcog1, Δmon1, Δccz1, Δpep7*

An important feature of the vacuole is its acidic pH. Vacuole acidification is regulated by a vacuolar-type ATPase (V-ATPase) protein complex. This protein complex is comprised of two multi subunits domains: a membrane integral V_0_ domain and a peripheral V_1_ domain [31]. Interaction between the two domains is essential for ATP-driven transport to maintain acidic vacuolar pH. Deletions of any of the eight subunits of the V1 complex were defective in generating H_2_S from cysteine (Fig. 5b) although these strains were able to grow under the experimental conditions (data not shown). Furthermore, deletion of genes encoding each of the three proteins responsible for V-ATPase assembly in the endoplasmic reticulum led to a similar phenotype of minimal H_2_S generation from cysteine (S1 Supporting Information). Interestingly, deletants for V_0_ subunits were able to generate H_2_S from cysteine supplemented medium at a rate similar to the wild-type strain.

The activity of yeast V-ATPase can be manipulated *in vivo* through growth medium composition [32]. When cells are transferred to a poor carbon source, up to 75% of existing V-ATPase complexes are disassembled into cytoplasmic (V_1_) and membrane-bound (V_o_) sectors, and this disassembly is completely reversible. We used this *in vivo* down-regulation mechanism to test whether V-ATPase assembly and activity is essential for cysteine degradation leading to H_2_S production or whether disassembled individual sectors have an effect on the process. H_2_S-producing cells grown on glucose were transferred into a growth medium containing glucose or galactose as sole carbon source. Fig. 5c shows that upon transfer to galactose-containing medium cells did not generate H_2_S, whereas those transferred to glucose-containing medium continued to produce H_2_S. Addition of 0.112 M glucose to the galactose medium restored H_2_S formation (Fig. 5c). In all, these findings are consistent with poor carbon source driven disassembly of the V-ATPase complex preventing cysteine catabolism to release H_2_S.

## Discussion

Cysteine is maintained at a low intracellular concentration by regulation of its production and, when in excess, its efficient removal. Amongst cysteine catabolic pathways is cysteine desulfhydration in which sulfur is cleaved from cysteine to produce H_2_S. In mammals, desulfhydration of cysteine leads to the production of H_2_S, which, in turn, may impact on cellular functions [1]. The current study used a EUROSCARF *S. cerevisiae* genome-wide deletion library for a functional genomics approach to identifying genes and cellular processes associated with the release of H_2_S from cysteine.

*S. cerevisiae* deletion libraries have been applied in a broad range of studies on biological processes including cellular response to Na^+^ [33], metal toxicity [34], oxidative stress [35], GSH homeostasis [36] and H_2_S formation through the sulfate assimilation pathway [37]. The latter utilised BiGGy agar [38] as an indicator for sulfite reductase activity, with follow up fermentation experiments on selected strains using gas collection tubes [39]. In this study, screening of a haploid yeast deletion library revealed a large number of genes (226) whose deletion altered H_2_S formation via cysteine catabolism. The caveat to this genetic approach is the relatively high level of H_2_S production from cysteine observed for the wild-type strain, which is close to the method's upper detection limit (Fig. 1). For this reason, the screen was aimed more at the identification of mutants impaired in H_2_S-generating catabolism of cysteine and discoveries made here regarding high H_2_S producers require further validation. An additional consideration is the genetic background of strain BY4742 that includes a number of auxotrophies. The possibility that discoveries observed here may be influenced by the genetic background of this deletion collection must be acknowledged.

Two PLP-dependent enzymes, highly conserved across prokaryotes and eukaryotes, were previously implicated in H_2_S-generating cysteine catabolism: CBS and CSE [1], [13], [14], [40]. The *S. cerevisiae* genes encoding these enzymes are *CYS4* and *CYS3*, respectively. In this genomic screen, deletion of *CYS4* did not affect H_2_S production from cysteine (S1 Supporting Information). Growth of a *Δcys3* strain was delayed under cysteine supplementation, however cells still reached stationary phase of growth and were able to catabolise cysteine to release H_2_S (S1 Supporting Information). These results are rather surprising considering these enzymes are central in cysteine catabolism to release H_2_S in higher eukaryotes. However yeast differ from other eukaryotes by having an additional pathway for H_2_S generation, through sulfate assimilation. It has been previously reported that deletion or inhibition of cys3/cys4 results in increased H_2_S formation through the sulfate assimilation pathway [41], [42], which may compensate for the decrease in cysteine catabolism to release H_2_S. The regulatory mechanism and relative contribution of the two enzymes to H_2_S generation and cysteine metabolism is not yet understood [40], [43] and further study is needed to understand the catalytic and regulatory role of these enzymes. Additionally, the possibility that other PLP-dependent enzymes play some role in this process must be considered.

### Cysteine in Fe-S cluster biogenesis

An additional PLP-dependent enzyme, Nfs1p, which is similar to its bacterial orthologs NifS and IscS, produces sulfur from cysteine for incorporation into Fe-S proteins [44], [45]. In eukaryotes, including *S. cerevisiae*, the biosynthesis of Fe-S clusters is mostly carried out in the mitochondrion [46]–[48]. Amongst other physiological roles, the synthesis of Fe-S clusters serves to maintain mitochondrial iron homeostasis [48]–[50]. Disruption of this process leads to mitochondrial iron overload, which is detrimental to the cell [48]. Accordingly, null mutation of many of the genes involved in Fe-S cluster biogenesis, including *NFS1*, results in cell death [28].

In this study mutants involved in all aspects of iron-sulfur homeostasis maintenance produced large amounts of H_2_S in comparison to the wild type strain (Fig. 3A). Increased production of H_2_S was also observed in a respiratory deficient petite mutant (Fig. 3B), although these mutants produced more H_2_S than the wild-type cells even in the absence of exogenous cysteine. The link between mitochondrial function and H_2_S formation was observed in Linderholm et al. (2008) [37], where deletion of gene *YIA6*, involved in NAD^+^ transport to mitochondria, resulted in high H_2_S production when cells were grown on BiGGy agar. The fact that deletants involved in Fe-S cluster assembly were not identified as H_2_S over-producers in Linderholm et al. (2008) [37] suggests that this phenotype appears only when cysteine is in excess, and reinforces the connection between iron-sulfur homeostasis and cysteine catabolism. Taken together these results lend weight to the hypothesis that disruption of Fe-S clusters leads to increased sulfur release associated with cysteine catabolism. However, the downstream metabolism of H_2_S must be considered as well and further studies are needed to understand the mitochondria role in H_2_S biogenesis and clearance.

### Cysteine link to purine biosynthesis and folate derived one-carbon metabolism

Acting as a sulfur source for Fe-S biogenesis is one of the many cellular functions of cysteine. Cysteine is one of the twenty amino acids required for protein synthesis and is a precursor for synthesis of several other essential molecules including GSH, coenzyme A, taurine, and inorganic sulfur [1]. Additionally, cysteine concentration regulates transcription of genes associated with yeast sulfur assimilation and metabolism [41], [51]. A link between purine metabolism and H_2_S production was previously made by Linderholm et al. (2008) [37]. In that study *S. cerevisiae* mutants with deletions in genes involved in purine metabolism produced darker colour colonies when grown on BiGGY agar, indicative of high concentrations of H_2_S. Here, the same deletants produced less H_2_S than wild-type cells upon cysteine supplementation during fermentation. The difference between the two results may simply reflect the different pathways examined; Linderholm *et al.* (2008) [37] studied H_2_S formation through the sulfate assimilation pathway while here the release of H_2_S from cysteine was studied. Alternatively, differences in the medium composition between the two experiments could have led to contrasting outcomes. In this study, addition of adenine resulted in increased H_2_S production, at a similar level to the wild-type strain (Fig. 4), suggesting the observed low H_2_S production was due to auxotrophic requirements of the deletants. Though this aspect was not tested in Linderholm, et al., (2008) [37] further studies are needed to identify the extent of a link between sulfur and purine metabolism.

A possible connection between the two is through folate derived one-carbon metabolism, necessary for synthesis of purines and methionine [30]. We report here, somewhat surprisingly, that disruption of methionine biosynthesis through the methyl cycle results in decreased catabolism of cysteine to release H_2_S, without affecting growth rate (Fig. 4B). Under these conditions one pathway for cysteine catabolism (conversion to methionine) is inhibited, thus the fact that cysteine was not catabolised to produce H_2_S under these conditions requires further investigation.

### Central role of the vacuole in cysteine catabolism

Cysteine accumulation was previously shown to have a cytotoxic effect [2], [3]. With that consideration, the central role discovered here for the vacuole in catabolism of cysteine to release H_2_S is reasonable. The yeast vacuole is an acidic compartment that shares a great deal of functional and morphological similarity with the mammalian lysosome and supports detoxification, protein degradation and ion and metabolite storage [52], [53]. Our genome-wide screen identified a strong link between the vacuole and degradation of cysteine to release H_2_S (Fig. 5). Of particular importance, deletion of each of the subunits comprising the V_1_ sub complex of V-ATPase, responsible for vacuolar acidic pH, resulted in low (to undetected) H_2_S production from cysteine. V-ATPases are evolutionary conserved multisubunit enzymes responsible for acidification of the vacuole in yeast and the lysosome in mammals [53]. Deletion of genes encoding ubiquitous V-ATPase subunits is lethal in all eukaryotes except fungi [53], due to their ability to take proton from the extracellular environment and thus maintain acidic intracellular pH [53]. Therefore, yeast impaired in V-ATPase activity are able to grow at a similar rate to the wild type under acidic conditions (pH<5), making studies of this complex in *S. cerevisiae* particularly important. Serendipitously, the medium used for our experiments was at pH 4.5, based on the optimal pH needed for H_2_S detection using the MBR method [22]. It is possible that the role of V-ATPase in H_2_S formation from cysteine is to encourage conversion of HS to H_2_S, downstream of enzymatic release from cysteine. This aspect requires further investigation.

Observations made in this study infer a major role of V-ATPase in the catabolism of excess cysteine to H_2_S and highlight the peripheral V_1_ sub-complex as the active unit. Most interestingly, deletants impaired in V-ATPase activity were previously classified as high H_2_S producers when evaluated for sulfate reductase activity using BiGGy agar [37]. While their classification was not validated, the observations differ from those described in this study possibly due to the pleiotropy of the V-ATPase. Impaired V-ATPase activity has been reported from studies utilising genomic screens to assess sensitivity to various drugs, metal ions, multiple forms of oxidative and other stresses (these studies are summarised in [54]. This suggests multiple roles for the V-ATPase complex, depending on the conditions under which yeast is grown. It is therefore plausible that activation of the sulfate reduction pathway in these deletants promotes high H_2_S production through a mechanism unrelated to cysteine catabolism. As the use of different environmental conditions leads to different H_2_S production patterns [22], the role of yeast V-ATPase should be evaluated under these environmental conditions using a yeast strain that is able to produce large amounts of H_2_S through both sulfate reduction and cysteine catabolism, possibly utilising labelled sulfur sources.

Regardless, the nature of the role of V-ATPase in cysteine catabolism requires further elucidation. V-ATPase generates a proton gradient across the vacuolar membrane that drives transport of ions and small molecules into the vacuole [55], [56]. It is possible that cysteine transport into the vacuole is facilitated by this proton gradient, which would explain our identification of the proton-generating ATP hydrolysis sub-complex (V_1_) as the active contributor to cysteine degradation. Supporting this, GSH transport to the vacuole is also partially mediated through a V-ATPase coupled system [57]. Alternatively, the observation that deletants impaired in vesicle formation and fusion with the vacuole (Table 1) are impaired in cysteine catabolism to release H_2_S, suggests that V-ATPase activity may facilitate cysteine transport to the vacuole in a vesicle-mediated manner. This is in line with recent studies demonstrating vesicle fusion with the vacuole requires the activity of the V-ATPase complex [53], [58]. Interestingly, cysteine metabolism was indirectly linked with V-ATPase, through the discovery that *Δcys4* mutants display an *in vivo* loss of vacuole acidification, due to inactivation of the V-ATPase complex [59]. It has been suggested that V-ATPase activity is regulated by cytosolic redox state; a concept supported by other studies from which it has been proposed that the thiol/disulphide ratio may serve as a “third messenger” [60]. These findings indicate that, in addition to being regulated by redox state, V-ATPase may operate to maintain cytosolic redox balance by removing excess cysteine from the cytosolic pool.

### Cysteine catabolism as a detoxification mechanism

Incorporation into GSH must be considered as a potential mechanism of cysteine exclusion from the cytosolic pool. GSH synthesis facilitates cysteine homeostasis by acting as the cellular reservoir for this amino acid. However, GSH homeostasis is tightly regulated [36] and the capacity for it to act as cysteine reservoir is limited. Thus it is unlikely to be the main destination in cysteine exclusion from the cytosolic pool. Supporting this view we observed catabolism of cysteine to release H_2_S in mutants impaired in GSH synthesis (*Δgsh1*, *Δgsh2*), at a similar level to the wild-type.

Two genome-wide studies have explored GSH homeostasis, analysing both its intra- and extracellular concentration [36], [61]. Our findings suggest that deletants with perturbed cysteine catabolism generally differ from those affecting GSH homeostasis. Most notably, there was considerable similarity between our findings and results from genome-wide screens for metal tolerance [34], [62]; deletants impaired in V-ATPase activity are sensitive to metal toxicity and have compromised cysteine catabolism leading to release of H_2_S. In addition, mutants reported to confer metal resistance were similar to those conferring high H_2_S production. These points of similarity reinforce that degradation of excess cysteine to H_2_S is a detoxification mechanism.

### Conclusions

Cysteine catabolism generates reduced sulfur and is critical for cysteine homeostasis in eukaryotes [1]. There is a substantial body of evidence supporting the physiological roles of cysteine degradation to release H_2_S, highlighting the need for a better understanding of the genetic factors influencing this part of metabolism. In this study we identified genes associated with cysteine catabolism that have not been previously linked with this process. The vacuolar role that has been established here and particularly the role of yeast V-ATPase point to new directions for future studies in cysteine homeostasis using *S. cerevisiae* as a model organism.

## Acknowledgments

We thank Laffort Australia and in particular Dr. Tertius Van der Westhuizen for continued support. Drs Paul Chambers and Paul Henschke are thanked for critical review of the manuscript. Jane McCarthy is thanked for organisation of the yeast deletion collection. Jenny Bellon is acknowledged for preparation of respiratory deficient petite yeast cells and Chiara Bozzinni for assistance with shake-flask H_2_S profile generation. We thank Angus Forgan and Dr Simon Schmidt for their help with programming of robotics for high-throughput screening experiments. The research was supported by an Industry Partnership grant. Research at The Australian Wine Research Institute is supported by Australia's grapegrowers and winemakers through their investment agency the Grape and Wine Research and Development Corporation, with matching funds from the Australian Government. The Australian Wine Research Institute is a member of the Wine Innovation Cluster.

## Supporting Information

S1 Supporting Information **Genes classified as low or high H2S producers in a genome-wide screen for cysteine catabolism.**
(DOCX)

S2 Supporting Information **H_2_S accumulation during growth of selected deletants classified as high/low H_2_S producers.** H_2_S accumulation in wild type (white column) and selected deletants classified as high (red columns: *Δfra1, Δisu1, Δmrs3, Δmtm1, Δisa1*) or low (green columns: *Δvma5, Δvam7, Δvam1, Δshm2*) H_2_S producers following cysteine catabolism to release H_2_S as measured after 16 hours of cultivation when it plateaued. Culture medium was based on [22] without the addition of sulfate and with supplementation of 500 mg/L cysteine. Strains cultivation and H2S measurements are described in Materials and Methods section. Error bars represent standard deviation of triplicate experiments and different letters denote significance at p<0.05 (Tuckey test).
(DOCX)
