# Supplementary material for: Formation of Hydrogen Sulfide from Cysteine in Saccharomyces cerevisiae BY4742: Genome Wide Screen Reveals a Central Role of the Vacuole
Source: PLoS One. 2014 Dec 17;9(12):e113869. doi: 10.1371/journal.pone.0113869 (PMC4269451; doi:10.1371/journal.pone.0113869)
Supplement: S1 Supporting Information — Genes classified as low or high H2S producers in a genome-wide screen for cysteine catabolism. (DOCX) [file pone.0113869.s001.docx]

**Supporting Information S1- Genes classified as low or high H_2_S producers in a genome-wide screen for cysteine catabolism**

| **ORF** | **Name** | **PHENOTYPE** |
| --- | --- | --- |
| YNL141W | AAH1 | LOW |
| YOR239W | ABP140 | MODERATELY LOW |
| YLR144C | ACF2 | MODERATELY HIGH |
| YAR015W | ADE1 | LOW |
| YOR128C | ADE2 | LOW |
| YGL234W | ADE5,7 | LOW |
| YGR061C | ADE6 | LOW |
| YDR408C | ADE8 | LOW |
| YJR105W | ADO1 | LOW |
| YBL082C | ALG3 | MODERATELY LOW |
| YFL050C | ALR2 | MODERATELY LOW |
| YDR441C | APT2 | LOW |
| YGL148W | ARO2 | LOW |
| YPR060C | ARO7 | LOW |
| YJL115W | ASF1 | LOW |
| YGR124W | ASN2 | MODERATELY LOW |
| YFR021W | ATG18 | MODERATELY LOW |
| YIL088C | AVT7 | LOW |
| YKR099W | BAS1 | MODERATELY LOW |
| YLR399C | BDF1 | HIGH |
| YIL159W | BNR1 | HIGH |
| YGL007W | BRP1 | LOW |
| YCR063W | BUD31 | MODERATELY LOW |
| YDL099W | BUG1 | MODERATELY HIGH |
| YJL209W | CBP1 | LOW |
| YAL021C | CCR4 | LOW |
| YGL003C | CDH1 | MODERATELY LOW |
| YGL029W | CGR1 | LOW |
| YMR198W | CIK1 | MODERATELY HIGH |
| YGL223C | COG1 | LOW |
| YLR201C | COQ9 | HIGH |
| YBR036C | CSG2 | MODERATELY LOW |
| YBR131W | CVT16 | LOW |
| YGL078C | DBP3 | LOW |
| YKR024C | DBP7 | LOW |
| YER124C | DSE1 | MODERATELY LOW |
| YER176W | ECM32 | MODERATELY LOW |
| YNL084C | END3 | MODERATELY LOW |
| YNL280C | ERG24 | MODERATELY HIGH |
| YLR342W | FKS1 | LOW |
| YER182W | FMP10 | HIGH |
| YBR047W | FMP23 | LOW |
| YLL029W | FRA1 | HIGH |
| YGL220W | FRA2 | HIGH |
| YAL028W | FRT2 | MODERATELY LOW |
| YBR021W | FUR4 | LOW |
| YOR183W | FYV12 | MODERATELY LOW |
| YNL133C | FYV6 | MODERATELY LOW |
| YLR068W | FYV7 | LOW |
| YNL199C | GCR2 | MODERATELY LOW |
| YDR507C | GIN4 | LOW |
| YGR163W | GTR2 | MODERATELY LOW |
| YDL234C | GYP7 | LOW |
| YKL109W | HAP4 | MODERATELY HIGH |
| YOL095C | HMI1 | MODERATELY HIGH |
| YDR174W | HMO1 | LOW |
| YDR158W | HOM2 | LOW |
| YBR133C | HSL7 | LOW |
| YDR533C | HSP31 | LOW |
| YCR071C | IMG2 | MODERATELY HIGH |
| YIL002C | INP51 | MODERATELY LOW |
| YDR315C | IPK1 | LOW |
| YLL027W | ISA1 | HIGH |
| YPL135W | ISU1 | HIGH |
| YDL115C | IWR1 | MODERATELY HIGH |
| YGL173C | KEM1 | LOW |
| YAR018C | KIN3 | MODERATELY LOW |
| YOR322C | LDB1 | MODERATELY LOW |
| YFR001W | LOC1 | LOW |
| YFL018C | LPD1 | LOW |
| YOR084W | LPX1 | MODERATELY HIGH |
| YJL124C | LSM1 | LOW |
| YKL176C | LST4 | LOW |
| YAL024C | LTE1 | LOW |
| YCR020C-A | MAK31 | MODERATELY LOW |
| YKR007W | MEH1 | LOW |
| YOL064C | MET22 | MODERATELY LOW |
| YDL200C | MGT1 | LOW |
| YDR031W | MIC14 | LOW |
| YDR245W | MNN10 | LOW |
| YJL183W | MNN11 | LOW |
| YGL124C | MON1 | LOW |
| YNL284C | MRPL18 | HIGH |
| YKR085C | MRPL20 | HIGH |
| YMR193W | MRPL24 | LOW |
| YLR439W | MRPL4 | HIGH |
| YGR165W | MRPS35 | HIGH |
| YJL133W | MRS3 | HIGH |
| YGR257C | MTM1 | HIGH |
| YPL226W | NEW1 | MODERATELY LOW |
| YOR209C | NPT1 | MODERATELY LOW |
| YHL029C | OCA5 | LOW |
| YGL038C | OCH1 | LOW |
| YKL134C | OCT1 | HIGH |
| YOR036W | PEP12 | LOW |
| YDR079W | PET100 | HIGH |
| YBR168W | PEX32 | MODERATELY LOW |
| YOL136C | PFK27 | MODERATELY HIGH |
| YDL236W | PHO13 | LOW |
| YBR092C | PHO3 | MODERATELY HIGH |
| YBR106W | PHO88 | MODERATELY LOW |
| YGL023C | PIB2 | MODERATELY LOW |
| YHR034C | PIH1 | LOW |
| YDR490C | PKH1 | MODERATELY LOW |
| YGR123C | PPT1 | MODERATELY LOW |
| YML016C | PPZ1 | MODERATELY LOW |
| YOR323C | PRO2 | MODERATELY LOW |
| YML017W | PSP2 | MODERATELY LOW |
| YKL076C | PSY1 | MODERATELY LOW |
| YDR496C | PUF6 | MODERATELY LOW |
| YGL058W | RAD6 | MODERATELY LOW |
| YGL246C | RAI1 | LOW |
| YPL246C | RBD2 | MODERATELY HIGH |
| YOR107W | RGS2 | HIGH |
| YHL027W | RIM101 | MODERATELY HIGH |
| YGL046W | RIM8 | HIGH |
| YEL050C | RML2 | HIGH |
| YGL250W | RMR1 | LOW |
| YGR180C | RNR4 | MODERATELY LOW |
| YPL123C | RNY1 | LOW |
| YBR084C-A | RPL19A | MODERATELY LOW |
| YBL027W | RPL19B | LOW |
| YBL087C | RPL23A | LOW |
| YER117W | RPL23B | MODERATELY LOW |
| YGL031C | RPL24A | MODERATELY LOW |
| YHR010W | RPL27A | LOW |
| YFR032C-A | RPL29 | LOW |
| YFR031C-A | RPL2A | LOW |
| YDL191W | RPL35A | LOW |
| YDR500C | RPL37B | MODERATELY LOW |
| YLR448W | RPL6B | MODERATELY LOW |
| YGL076C | RPL7A | LOW |
| YHL033C | RPL8A | MODERATELY LOW |
| YGL147C | RPL9A | LOW |
| YDR382W | RPP2B | LOW |
| YMR230W | RPS10B | LOW |
| YGR027C | RPS25A | LOW |
| YOR182C | RPS30B | MODERATELY LOW |
| YBR181C | RPS6B | MODERATELY LOW |
| YBL072C | RPS8A | MODERATELY LOW |
| YHR021C | RPSD27B | LOW |
| YIL153W | RRD1 | LOW |
| YHR038W | RRF1 | LOW |
| YGL244W | RTF1 | HIGH |
| YOR014W | RTS1 | LOW |
| YKL212W | SAC1 | LOW |
| YOR184W | SER1 | MODERATELY LOW |
| YLR058C | SHM2 | LOW |
| YBL058W | SHP1 | LOW |
| YMR216C | SKY1 | LOW |
| YBL007C | SLA1 | LOW |
| YOR008C | SLG1 | LOW |
| YBR266C | SLM6 | LOW |
| YBR172C | SMY2 | LOW |
| YNR023W | SNF12 | LOW |
| YGL127C | SOH1 | HIGH |
| YCL048W | SPS22 | MODERATELY LOW |
| YBR081C | SPT7 | LOW |
| YHR066W | SSF1 | LOW |
| YLR362W | STE11 | HIGH |
| YLR372W | SUR4 | LOW |
| YHR009C | TDA3 | MODERATELY HIGH |
| YGR192C | TDH3 | MODERATELY LOW |
| YDR438W | THI74 | LOW |
| YPR074C | TKL1 | LOW |
| YER113C | TMN3 | MODERATELY LOW |
| YER090W | TRP2 | LOW |
| YOR006C | TSR3 | LOW |
| YLR425W | TUS1 | MODERATELY LOW |
| YOR344C | TYE7 | LOW |
| YDR207C | UME6 | MODERATELY LOW |
| YGL212W | VAM7 | LOW |
| YBR105C | VID24 | MODERATELY HIGH |
| YNL212W | VID24 | MODERATELY LOW |
| YLR410W | VIP1 | LOW |
| YGL212W | VAM7 | LOW |
| YHR039C-B | VMA10 | LOW |
| YPR036W | VMA13 | MODERATELY LOW |
| YGR105W | VMA21 | LOW |
| YHR060W | VMA22 | LOW |
| YEL027W | VMA3 | LOW |
| YKL080W | VMA5 | LOW |
| YEL051W | VMA8 | LOW |
| YKL119C | VPH2 | LOW |
| YKR001C | VPS1 | LOW |
| YDR495C | VPS3 | LOW |
| YGL095C | VPS45 | LOW |
| YDR484W | VPS52 | LOW |
| YOR359W | VTS1 | LOW |
| YDL224C | WHI4 | LOW |
| YPL239W | YAR1 | MODERATELY LOW |
| YBL100C | YBL100C | LOW |
| YBR074W | YBR074W | MODERATELY LOW |
| YBR206W | YBR206W | HIGH |
| YBR285W | YBR285W | LOW |
| YER123W | YCK3 | MODERATELY LOW |
| YCL007C | YCL007C | LOW |
| YCR102C | YCR102C | LOW |
| YDL199C | YDL199C | LOW |
| YDL237W | YDL237W | LOW |
| YDR010C | YDR010C | MODERATELY LOW |
| YDR266C | YDR266C | LOW |
| YDR417C | YDR417C | LOW |
| YER186C | YER186C | LOW |
| YER187W | YER187W | MODERATELY LOW |
| YFL034W | YFL034W | LOW |
| YGR102C | YGR102C | LOW |
| YJL188C | YJL188C | LOW |
| YJR096W | YJR096W | LOW |
| YKL118W | YKL118W | LOW |
| YLR225C | YLR225C | HIGH |
| YOL036W | YOL036W | MODERATELY LOW |
| YOR139C | YOR139C | MODERATELY HIGH |
| YOR309C | YOR309C | MODERATELY LOW |
| YPL102C | YPL102C | MODERATELY LOW |
| YPR117W | YPR117W | MODERATELY LOW |
| YBR264C | YPT10 | LOW |
| YIR026C | YVH1 | LOW |

**2.6.2 Go terms associated with low H_2_S producers**

**
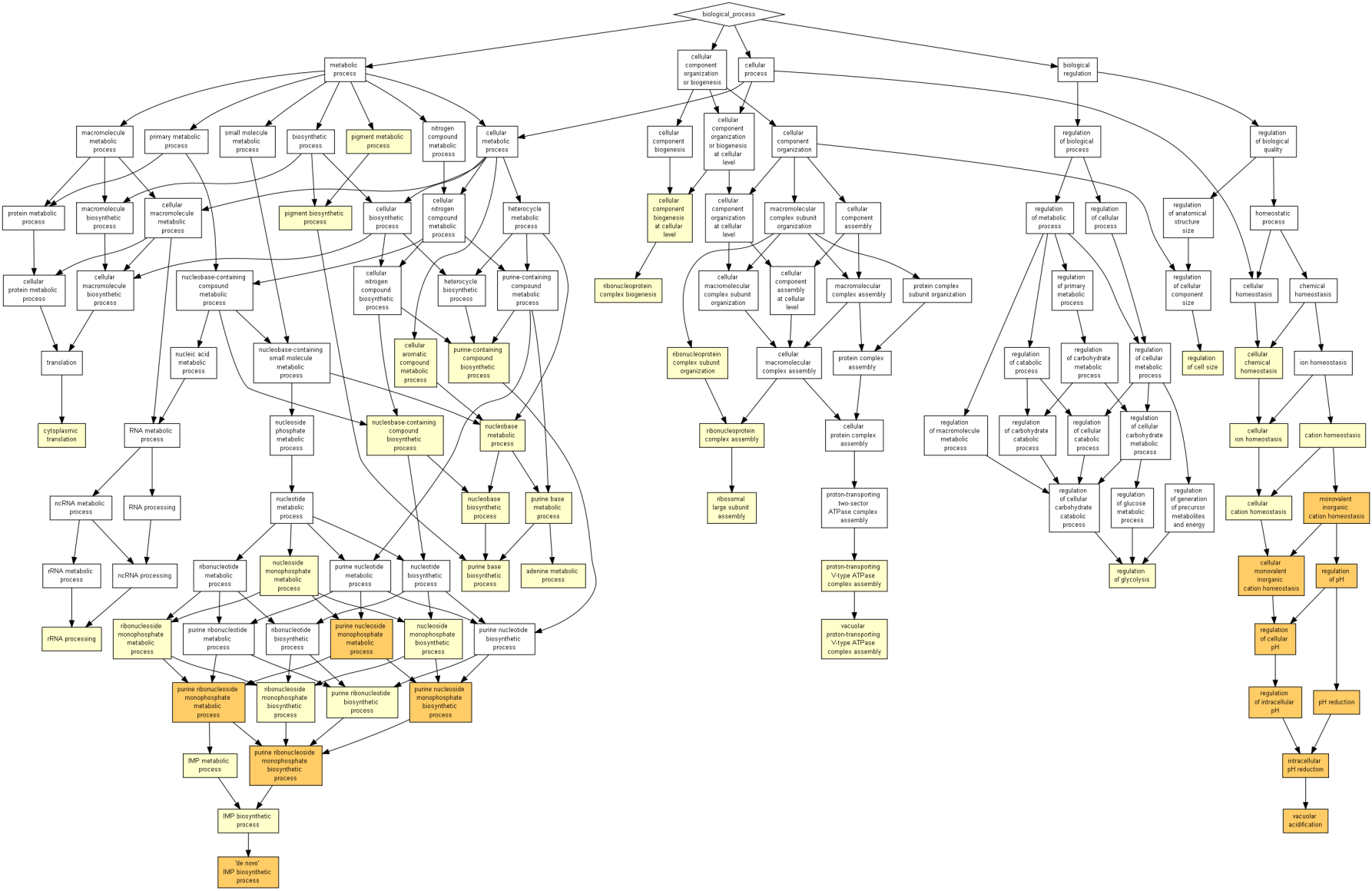
**

| **GO Term** | **Description** | **P-value** |
| --- | --- | --- |
| GO:0045851 | pH reduction | 2.12E-07 |
| GO:0007035 | vacuolar acidification | 2.12E-07 |
| GO:0051452 | intracellular pH reduction | 2.12E-07 |
| GO:0030641 | regulation of cellular pH | 3.19E-07 |
| GO:0051453 | regulation of intracellular pH | 3.19E-07 |
| GO:0030004 | cellular monovalent inorganic cation homeostasis | 5.99E-07 |
| GO:0006885 | regulation of pH | 9.69E-07 |
| GO:0055067 | monovalent inorganic cation homeostasis | 1.43E-06 |
| GO:0009168 | purine ribonucleoside monophosphate biosynthetic process | 3.85E-06 |
| GO:0009167 | purine ribonucleoside monophosphate metabolic process | 3.85E-06 |
| GO:0006189 | 'de novo' IMP biosynthetic process | 5.65E-06 |
| GO:0009126 | purine nucleoside monophosphate metabolic process | 5.88E-06 |
| GO:0009127 | purine nucleoside monophosphate biosynthetic process | 5.88E-06 |
| GO:0002181 | cytoplasmic translation | 1.15E-05 |
| GO:0006188 | IMP biosynthetic process | 1.23E-05 |
| GO:0046040 | IMP metabolic process | 1.23E-05 |
| GO:0006144 | purine base metabolic process | 2.49E-05 |
| GO:0009161 | ribonucleoside monophosphate metabolic process | 3.39E-05 |
| GO:0009156 | ribonucleoside monophosphate biosynthetic process | 3.39E-05 |
| GO:0009113 | purine base biosynthetic process | 4.21E-05 |
| GO:0009124 | nucleoside monophosphate biosynthetic process | 4.55E-05 |
| GO:0009123 | nucleoside monophosphate metabolic process | 7.83E-05 |
| GO:0030003 | cellular cation homeostasis | 9.02E-05 |
| GO:0000027 | ribosomal large subunit assembly | 1.10E-04 |
| GO:0022613 | ribonucleoprotein complex biogenesis | 1.57E-04 |
| GO:0071843 | cellular component biogenesis at cellular level | 2.06E-04 |
| GO:0006873 | cellular ion homeostasis | 2.59E-04 |
| GO:0006725 | cellular aromatic compound metabolic process | 2.62E-04 |
| GO:0070072 | vacuolar proton-transporting V-type ATPase complex assembly | 2.66E-04 |
| GO:0070070 | proton-transporting V-type ATPase complex assembly | 2.66E-04 |
| GO:0046083 | adenine metabolic process | 2.66E-04 |
| GO:0009112 | nucleobase metabolic process | 3.05E-04 |
| GO:0006364 | rRNA processing | 3.93E-04 |
| GO:0055082 | cellular chemical homeostasis | 4.17E-04 |
| GO:0072522 | purine-containing compound biosynthetic process | 4.78E-04 |
| GO:0055080 | cation homeostasis | 4.99E-04 |
| GO:0009152 | purine ribonucleotide biosynthetic process | 5.38E-04 |
| GO:0022618 | ribonucleoprotein complex assembly | 5.57E-04 |
| GO:0046148 | pigment biosynthetic process | 6.17E-04 |
| GO:0046112 | nucleobase biosynthetic process | 6.17E-04 |
| GO:0034654 | nucleobase-containing compound biosynthetic process | 6.27E-04 |
| GO:0006110 | regulation of glycolysis | 6.45E-04 |
| GO:0071826 | ribonucleoprotein complex subunit organization | 7.44E-04 |
| GO:0008361 | regulation of cell size | 7.89E-04 |
| GO:0042440 | pigment metabolic process | 8.10E-04 |

****** Strains were grouped according to the gene ontology description of the encoded gene product, as defined in the *Saccharomyces* Genome Database ([www.yeastgenome.org](http://www.yeastgenome.org)) using Gorilla ([Eden*, et al.*, 2009](#_ENREF_42)).

**2.6.2 GO terms associated with high H_2_S producers**

**
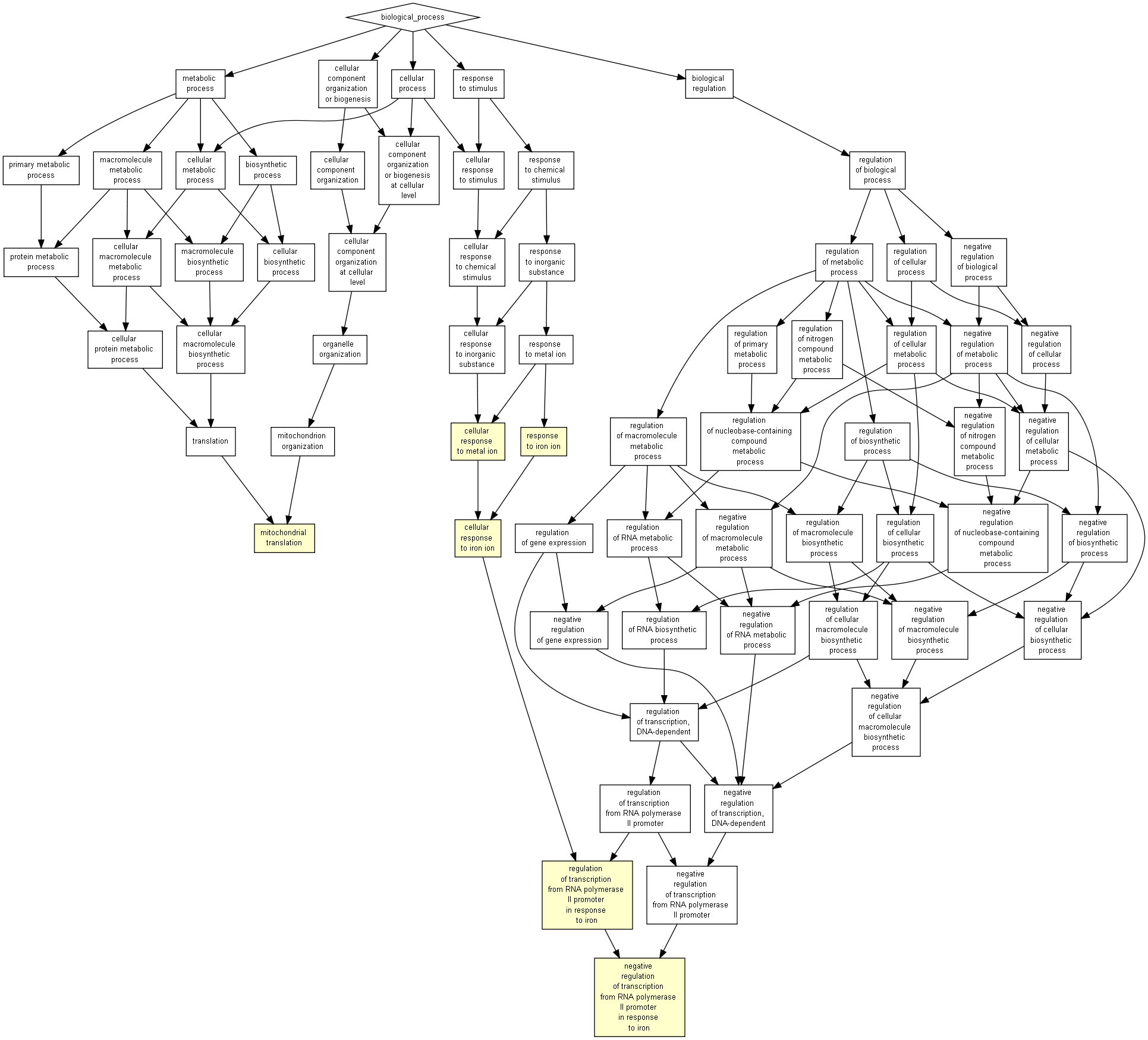
**

| **GO Term** | **Description** | **P-value** |
| --- | --- | --- |
| GO:0034396 | negative regulation of transcription from RNA polymerase II promoter in response to iron | 6.35E-05 |
| GO:0010039 | response to iron ion | 1.90E-04 |
| GO:0034395 | regulation of transcription from RNA polymerase II promoter in response to iron | 1.90E-04 |
| GO:0071281 | cellular response to iron ion | 1.90E-04 |
| GO:0032543 | mitochondrial translation | 3.75E-04 |
| GO:0071248 | cellular response to metal ion | 6.26E-04 |

**Strains were grouped according to the gene ontology description of the encoded gene product, as defined in the *Saccharomyces* Genome Database ([www.yeastgenome.org](http://www.yeastgenome.org)) using Gorilla ([Eden*, et al.*, 2009](#_ENREF_42)).
