# Supplementary figures and images for: Formation of Hydrogen Sulfide from Cysteine in Saccharomyces cerevisiae BY4742: Genome Wide Screen Reveals a Central Role of the Vacuole
Source: PLoS One. 2014 Dec 17;9(12):e113869. doi: 10.1371/journal.pone.0113869 (PMC4269451; doi:10.1371/journal.pone.0113869)

**Supporting Information S2**


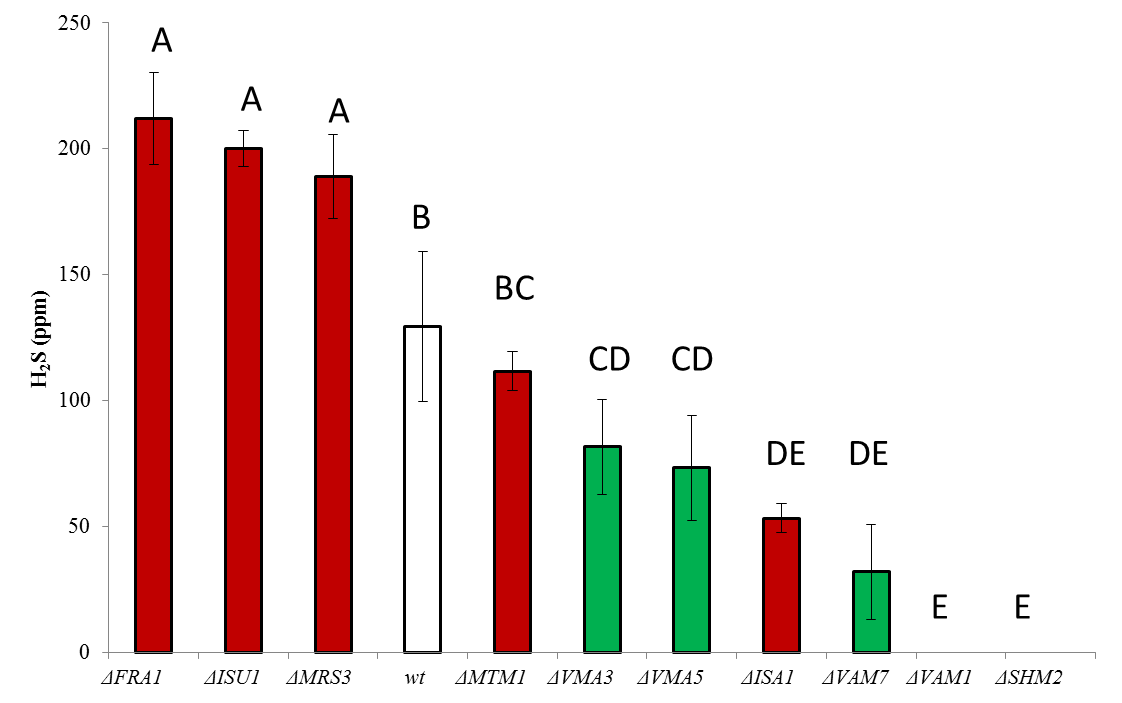

Supplement: S2 Supporting Information — H2S accumulation during growth of selected deletants classified as high/low H2S producers. H2S accumulation in wild type (white column) and selected deletants classified as high (red columns: Δfra1, Δisu1, Δmrs3, Δmtm1, Δisa1) or low (green columns: Δvma5, Δvam7, Δvam1, Δshm2) H2S producers following cysteine catabolism to release H2S as measured after 16 hours of cultivation when it plateaued. Culture medium was based on [22] without the addition of sulfate and with supplementation of 500 mg/L cysteine. Strains cultivation and H2S measurements are described in Materials and Methods section. Error bars represent standard deviation of triplicate experiments and different letters denote significance at p<0.05 (Tuckey test). (DOCX) [file pone.0113869.s002.docx]
